# Biased attention near another's hand following joint action

**DOI:** 10.3389/fpsyg.2013.00443

**Published:** 2013-07-22

**Authors:** Hsin-Mei Sun, Laura E. Thomas

**Affiliations:** Center for Visual and Cognitive Neuroscience, Department of Psychology, North Dakota State UniversityFargo, ND, USA

**Keywords:** spatial attention, hand position, perihand space, joint action, body schema, body representation

## Abstract

Previous research has shown that attention is prioritized for the space near the hand, leading to faster detection of visual targets appearing close to one's own hand. In the present study, we examined whether observers are also facilitated in detecting targets presented near another's hand by having participants perform a Posner cueing task while sitting next to a friend. Across blocks, either the participant or the friend placed a hand next to one of the target locations. Our results robustly showed that participants detected targets appearing near their own hands more quickly than targets appearing away from their hands, replicating previous work demonstrating that spatial attention is prioritized near one's own hand (Experiments 1–4). No such attentional bias effects were found for targets appearing near the friend's hand, suggesting that spatial attention is not automatically prioritized near another's hand (Experiments 1 and 2). However, participants were faster to detect targets near the friend's hand following a joint action task, suggesting a shared body representation plays an influential role in biasing attention to the space near another's hand (Experiment 4).

## Introduction

When observers position their hands near a visual display, they experience a variety of changes in visual and cognitive processing such as altered perception (Cosman and Vecera, 2010), memory (Tseng and Bridgeman, 2011), and semantic processing (Davoli et al., 2010). The presence of the hands also influences attentional processing, prolonging visual search (Abrams et al., 2008), delaying switches between processing global vs. local aspects of hierarchical figures (Davoli et al., 2012), and biasing the allocation of spatial attention to locations near the hands (Reed et al., 2006, 2010). In addition, observers suffering from visual neglect experience an attenuation of symptoms when they place a hand in the affected visual field (e.g., di Pellegrino and Frassinetti, 2000). This near-hand attentional facilitation effect may reflect a system of bimodal neurons responding to tactile and visual stimuli presented near the hand that strengthens visual processing of objects in perihand space (Graziano et al., 1997; di Pellegrino and Frassinetti, 2000; Schendel and Robertson, 2004; Reed et al., 2006), prioritizing processing of items near the hand that are candidates for future actions (Abrams et al., 2008; Cosman and Vecera, 2010; Reed et al., 2010; Tseng and Bridgeman, 2011).

Although the recent influx of research investigating vision near the hands clearly demonstrates that observers process and represent objects viewed near their own hands in a specialized manner, little work has examined whether the visual system might also prioritize visual information presented near the hands of another actor. Others' hands hold a special social significance. People often use their hands to direct others' attention through pointing and gesturing, creating a joint focus of attention (e.g., Bangerter, 2004). Observers seem to automatically process deictic gestures, taking in information about the direction of another individual's social attention (Langton et al., 1996; Langton and Bruce, 2000). In addition, when an individual sees another actor perform an action, neurons representing that action become active in the observer's motor cortex (e.g., Gallese et al., 1996). This automatically activated motor representation of the observed action closely corresponds to the representation generated when the observer executes the same action (e.g., Iacoboni et al., 1999). These shared representations of observed and executed actions play an influential role in action recognition, action imitation, and the ability to understand the intentions associated with the actions of others (Decety and Grezes, 1999; Blakemore and Decety, 2001; Rizzolatti et al., 2001; Buccino et al., 2004; Rizzolatti and Craighero, 2004; Iacoboni et al., 2005). In addition, observers employ similar neural mechanisms to monitor their own and others' task performance (van Schie et al., 2004) and integrate the potential acts of others into their own action plans (Sebanz et al., 2003, 2005; Atmaca et al., 2008). When individuals must work together to perform a task, they perceive object affordances based not on their own solo capabilities, but on what they can accomplish with their partner (e.g., Marsh et al., 2006). Observers also represent objects in terms of their affordances even when these objects are outside of their own reaching space, but remain in the reaching space of another actor (Costantini et al., 2011; Cardellicchio et al., 2013). Such findings suggest that observers map space not only in terms of their own action affordances, but also based on the potential of others to act on the environment.

Given the importance of others' hands in directing social attention and the significant role that shared representations play in action understanding and execution, it is possible that observers may experience changes in visual processing near the hands of other actors. We investigate whether observers represent the space near another person's hands in the same biased way they represent the space near their own hands. If the visual system prioritizes information presented near the hands of another actor, then observers should show biases in visual processing not only near their own hands, but also near the hands of others.

To test the hypothesis that the presence of another's hands influences visual processing, we asked participants to perform a covert attention task previously employed in an early study on the effects of hand positioning on visual attention (Reed et al., 2006). In the original work, participants detected a peripheral target appearing to the left or right of a central fixation cross after a highly predictive visual cue. In some conditions, participants placed one of their hands next to one of the target locations. Reed et al. (2006) found that, regardless of cue validity, participants were faster to detect targets appearing near their hands than targets appearing away from their hands, suggesting that participants prioritized attention to the space near their own hands. We used the same paradigm, but asked participants to perform this orienting task while sitting next to a friend. Across blocks, either the participant or the friend placed a hand next to one of the target locations. We were interested in whether the presence of another person's hand would facilitate observers' target detection performance in the same manner as their own hand. In Experiment 1, we examined whether spatial attention is prioritized to the space near one's own as well as another person's hand. In Experiment 2, we investigated the influence of visual similarity between one's own and another's hand on the allocation of attention near the hands. In Experiment 3, we explored how observers allocate attention to the space near a fake hand. Finally, in Experiment 4, we examined the influence of a joint action task on attentional prioritization of the space near the hands of another actor. To preview our results, we found that although participants were consistently facilitated in detecting targets appearing near their own hands or a fake hand corresponding to their own, they only showed an attentional bias near their friends' hands following a joint action task. These findings suggest that the presence of hands only influences visual processing when these hands are incorporated into the observer's own body schema.

## Experiment 1

The main purpose of Experiment 1 was to investigate whether participants' target detection performance in a Posner cueing task (Posner et al., 1987) would be affected by the presence of another person's hand. Although previous studies have shown that attention is prioritized for the space near the hand, leading to faster detection of visual targets appearing close to one's own hand (Reed et al., 2006, 2010), it is unclear whether observers would also be facilitated in detecting targets near another person's hand. If observers automatically prioritize the space near another's hand, participants should detect targets more quickly when they appear near another person's hand than when they appear away from another's hand. However, if the mere presence of another's hand does not lead to a default attentional bias, then the positioning of another person's hand should have no influence on target detection performance.

### Method

#### Participants

Thirty-four right-handed North Dakota State University undergraduates (18 females; mean *age* = 19.03 years) participated in the study for course credit. Participants brought a friend of the same sex to the lab to sit next to them during the study. All participants had normal or corrected-to-normal vision and were naïve to the purpose of the study. The experimental protocol was approved by the North Dakota State University Institutional Review Board for the protection of human participants in research, and informed consent was obtained from all participants.

#### Apparatus

All stimuli were drawn in black against a white background on a 19-inch monitor with a refresh rate of 75-Hz and a display resolution of 1024 × 768 pixels. A chin rest was used to maintain a constant viewing distance of 50 cm. Responses were collected through the computer keyboard. When asked to place a hand on the computer screen, participants and their friends rested an elbow on folded towels on the table to minimize the discomfort associated with prolonged extension of the hand and arm. The experiment was programmed in MATLAB, using the Psychophysics Toolbox extensions (Brainard, 1997; Pelli, 1997).

#### Procedure and design

**Figure 1:**
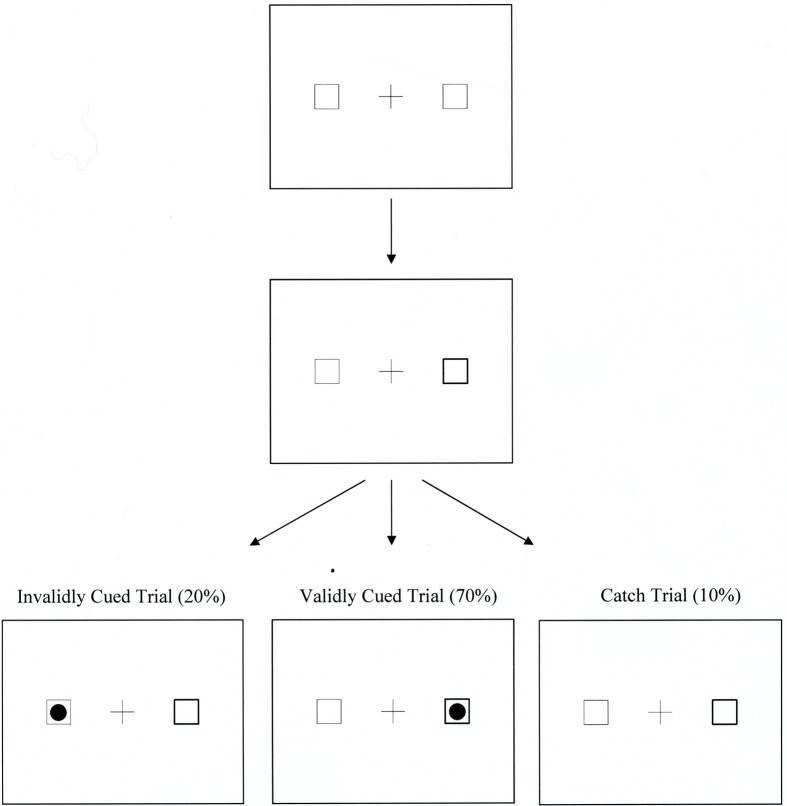
**The sequence of events within a trial in Experiments 1–4**.

Participants performed a covert attention task. During the task, they were presented with a central cross (3.4°), flanked by two empty squares (3.4°) located 7.4° to its left and right side. Participants were instructed to fixate the central cross on each trial. After a random delay lasting 1500–3000 ms, one of the peripheral squares was cued by increasing the thickness of its border. The target (a solid black dot; 2.2°) appeared 200 ms later in either the cued (valid trial) or uncued (invalid trial) square. There were also catch trials in which no target appeared in either square after a cue. Within each hand condition in the current study, 70% of the trials were validly cued, 20% of the trials were invalidly cued, and 10% were catch trials. Participants were instructed to press the space bar on the computer keyboard as soon as they detected the presence of the target and to refrain from responding on catch trials. For both valid and invalid trials, the cue and the target remained visible on the screen until the participant responded; for the catch trials, the cue stayed on the screen for 2000 ms and then disappeared. Figure 1 illustrates the sequence of events in a trial.

**Figure 2:**
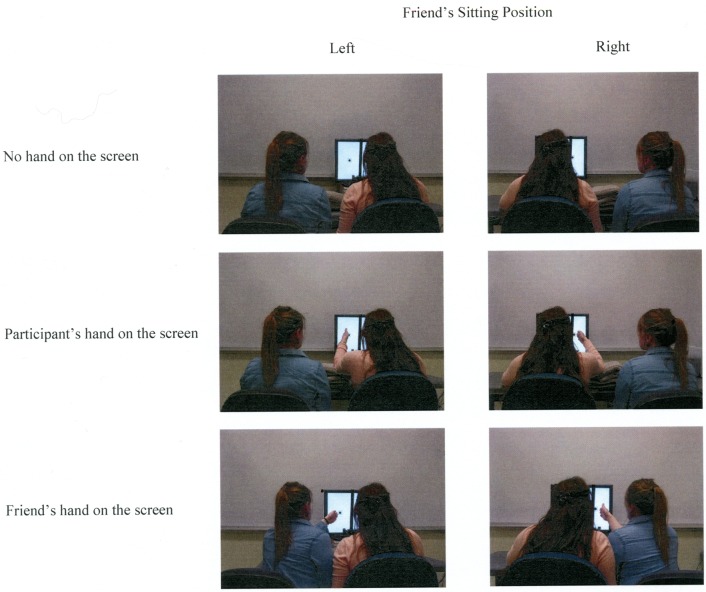
**Experimental setup and hand positions in Experiment 1**.

During the experiment, participants performed the covert attention task while sitting next to their friends. Participants' friends sat to their right for half of the experiment and to their left for the other half of the experiment in a counterbalanced order. Participants completed three experimental conditions for both positions. When the participant's friend sat on the right, the three experimental conditions were: (a) no hand on the screen, in which participants responded with the left hand and rested the right hand in the lap while the friend rested both hands in the lap; (b) participant's hand on the screen, in which participants responded with the left hand and placed their right hand next to the right square on the screen while the friend rested both hands in the lap; and (c) friend's hand on the screen, in which participants responded with the left hand and rested their right hand in the lap while the friend placed the right hand next to the right square on the screen. When the friend was sitting to the left-hand side of the participants, the three experimental conditions were the same, except that participants responded with their right hand, and participants or their friend placed the left hand next to the left square on the screen when required. For conditions in which a participant or friend placed a hand on the screen, the hand rested next to the outer-edge of the right/left square with the palm facing the square and the tip of the middle finger touching the computer screen. Before a block of trials in these conditions began, participants viewed a display with written instructions about hand placement, the empty squares and central cross, and one small filled dot (0.7°) placed 1.8° to the side of a square that served as a guide to help participants or their friend to position the hand in a consistent location on the display. The guide dot was removed before the first trial in a block began. Before each block of trials in the no hand conditions, participants viewed a display showing written instructions about hand placement as well as the empty boxes and central cross. Figure 2 shows the experimental settings and hand positions in the different experimental conditions for Experiment 1. There were two blocks of 60 trials for each condition for each friend's sitting position, resulting in a total of 12 total blocks—six blocks in which the friend sat on the participants' left and six blocks in which the friend sat on the participants' right. Block order was randomized. Prior to the formal sessions, participants completed a practice session of 20 trials in the no hand condition.

### Results and discussion

The dependent measure of interest in the current study was participants' reaction times (RTs) for target detection. Four participants made excessive catch trial response errors (>55%) and were eliminated from analyses. The remaining participants had an overall catch trial error rate of 16%. Data from trials in which participants responded outside a window of 200–1000 ms after the onset of the target (9% of total trials) were also eliminated from analyses to exclude errors of anticipation and inattention.

**Figure 3:**
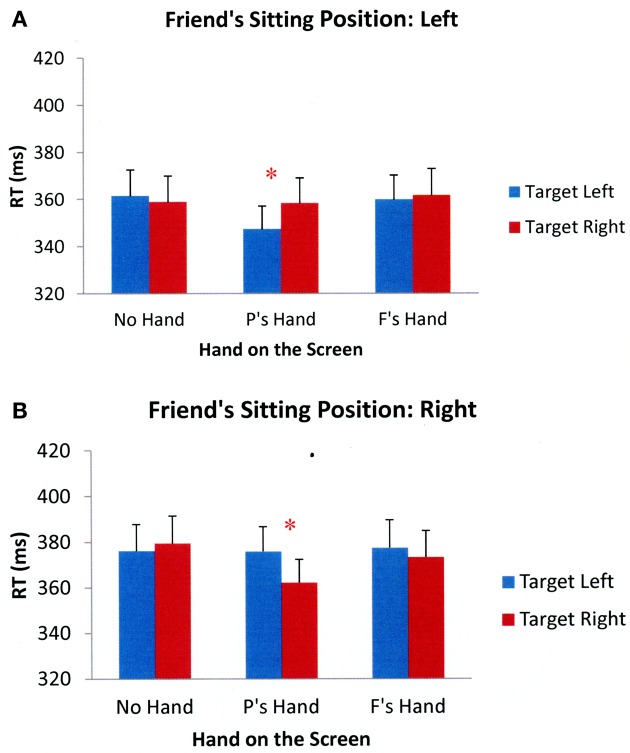
**Mean reaction times across all participants in different Experimental conditions of Experiment 1 when a friend was sitting to the participants' left (A) and right (B).** Error bars represent standard errors of the means. (Note: P's = Participant's; F's = Friend's). ^*^p < 0.05.

To examine participants' target detection performance, we conducted a 2 (friend's sitting position: left, right) × 3 (hand position: no hand on the screen, participant's hand on the screen, friend's hand on the screen) × 2 (target side: left, right) × 2 (cue validity: valid, invalid) repeated measures ANOVA. The results showed a significant main effect of the friend's sitting position, *F*_(1 29)_ = 4.765, *p* = 0.037, indicating that participants responded faster when their friend sat on their left- compared to right-hand side. This may be due to the fact that when a friend was sitting on participants' left-hand side, participants had to respond with their dominant hand (right hand) and thus produced faster response times^1^. Additionally, there was a significant main effect of cue validity, *F*_(1, 29)_ = 121.069, *p* < 0.001, showing that participants responded more quickly to validly compared to invalidly cued trials. Importantly, the three-way interaction between friend's sitting position, hand position, and target side was also significant, *F*_(2, 58)_ = 10.166, *p* < 0.001. There were no other significant main effects or interactions. As in previous research employing this paradigm, cue validity had no impact on near-hand effects, suggesting that hand presence did not influence the shifting of attention, but instead affected attentional prioritization of space (Reed et al., 2006, 2010). Figure 3 shows the mean reaction times across all participants in the different experimental conditions collapsed across cue validity.

Separate 3 (hand position: no hand on the screen, participant's hand on the screen, friend's hand on the screen) × 2 (target side: left, right) ANOVAs were conducted for each sitting position to further examine the three-way interaction effect. The results showed that when a friend sat on the left-hand side of the participants, there were no significant main effects of hand position, *F*_(2, 58)_ = 1.566, *p* = 0.218, or target side, *F*_(1, 29)_ = 0.741, *p* = 0.396. However, the interaction between hand position and target side was significant, *F*_(2, 58)_ = 3.796, *p* = 0.028. Paired-samples *t*-tests further showed that participants were faster to detect targets appearing next to their own hands than targets appearing away from their hands, *t*_(29)_ = −2.330, *p* = 0.027, but were no faster to detect targets on one side of the screen than another when their friend's hand was on the screen *t*_(29)_ = −0.410, *p* = 0.685, or when no hand was placed on the screen, *t*_(29)_ = 0.524, *p* = 0.604. The same results were obtained when a friend sat on the right-hand side of the participants. There were no significant main effects of hand position, *F*_(2, 58)_ = 1.781, *p* = 0.178, or target side, *F*_(1, 29)_ = 1.999, *p* = 0.168, but the interaction between hand position and target side was significant, *F*_(2, 58)_ = 4.498, *p* = 0.015. Paired-samples *t*-tests showed that participants responded faster to targets appearing next to their hand compared to targets appearing away from their hand, *t*_(29)_ = −2.783, *p* = 0.009, but again, there were no differences between target detection times when a friend's hand was placed on the screen, *t*_(29)_ = −1.026, *p* = 0.313, or when no hand was placed on the screen, *t*_(29)_ = 0.643, *p* = 0.525. Together, these results suggest that target detection was facilitated near participants' own hands, but not their friend's hands.

In sum, Experiment 1 demonstrated that participants' detection performance was better for targets appearing near their own hands compared to targets appearing away from their hands, replicating previous research showing that the presence of one's own hand affects attentional prioritization and results in faster target detection near the hand (e.g., Reed et al., 2006, 2010). However, the results of Experiment 1 showed that participants were no faster to detect targets near a friend's hand than targets away from a friend's hand. Therefore, although previous research has shown that observers' attention is sensitive to the signals generated by others' hands (e.g., Langton et al., 1996) and that observers represent the actions and affordances of others in the same way they represent their own (e.g., Sebanz et al., 2003; Costantini et al., 2011), our results indicate that observers do not automatically prioritize the space near a friend's hand during a covert attention task. The biases associated with visual processing near the hands may be unique to an observer's own hands.

## Experiment 2

The results of Experiment 1 showed that the mere presence of another's hand is not sufficient to bias participants' attention to the space near a hand that is not their own. These results suggest that only the presence of one's own hands will drive changes in visual processing. Yet previous research suggests that the presence of a fake hand can also alter vision: when participants wear a rubber glove that matches a glove on a fake hand positioned on a display, they are faster to detect targets appearing near the fake hand than targets appearing away from the fake hand (Reed et al., 2006). Presumably, the correspondence between the rubber glove on the false hand and the glove on the participants' own handled participants to prioritize the space near the fake hand. Perhaps observers would likewise prioritize attention near a friend's hand if there were sufficient correspondence between their own and their friends' hands. To examine this possibility, in Experiment 2, we replicated the design of Experiment 1 but increased the visual similarity between the participants' own hands and their friends' hands. Participants and their friends wore matching rubber gloves to increase the correspondence between hands. If the visual similarity between hands creates a correspondence that leads observers to prioritize the space near another's hand, participants should respond faster to targets appearing next to their friend's hand than targets appearing away from the hand.

### Method

#### Participants

Thirty-two right-handed undergraduates from North Dakota State University (17 females; mean age = 19.03 years), all with normal or corrected-to-normal vision, participated in the study for course credit with a friend of the same sex. Participants were naïve to the purpose of the study and had not participated in the previous experiment.

#### Apparatus, procedure, and design

Experiment 2 was identical to Experiment 1, except that we had participants and their friends put a rubber glove on the hand that had to be placed on the screen. That is, when a friend was sitting on the right-hand side of the participants, they had to put a rubber glove on their right hand, and vice versa. Therefore, four rubber gloves were used: two for participants' right and left hands, and two for their friends' right and left hands.

### Results and discussion

**Figure 4:**
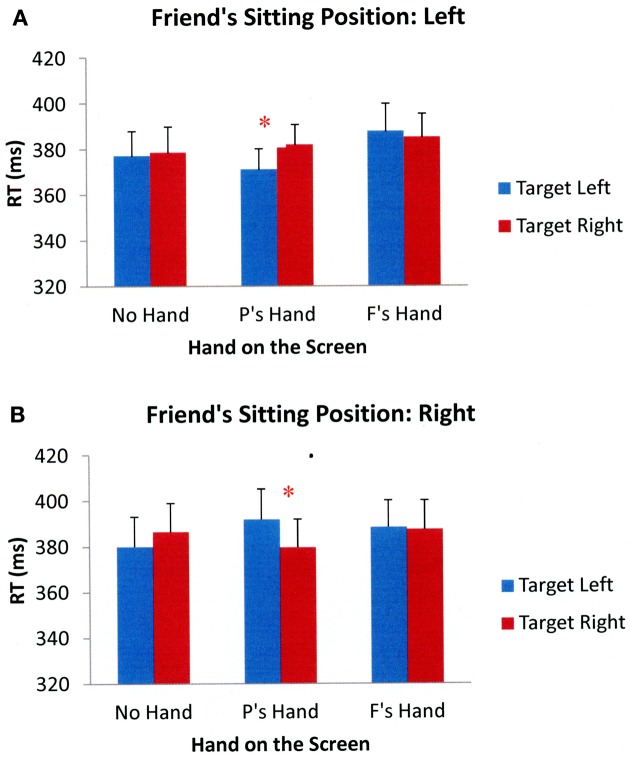
**Mean reaction times across all participants in different Experimental conditions of Experiment 2 when a friend was sitting to the participants' left (A) and right (B).** Error bars represent standard errors of the means. (Note: P's = Participant's; F's = Friend's). ^*^p < 0.05.

We used the same criteria as in Experiment 1 to analyze participants' RTs to detect the target. Four participants were excluded for excessive catch trial errors. The overall error rate for the remaining participants was 15% and another 8% of trials fell outside the window of 200–1000 ms^2^. The data were submitted to a 2 (friend's sitting position: left, right) × 3 (hand position: no hand on the screen, participant's hand on the screen, friend's hand on the screen) × 2 (target side: left, right) × 2 (cue validity: valid, invalid) repeated measures ANOVA. The results showed a significant main effect of cue validity, *F*_(1, 27)_ = 144.263, *p* < 0.001, demonstrating that participants were faster in responding to validly cued targets compared to invalidly cued targets. There was also a significant three-way interaction between friend's sitting position, hand position, and target side, *F*_(2, 54)_ = 6.825, *p* = 0.002. There were no other significant main effects or interactions. The mean RTs across participants in different experimental conditions collapsed across cue validity are shown in Figure 4.

To further examine the significant interaction between the friend's sitting position, hand position, and target side, separate 3 (hand position: no hand on the screen, participant's hand on the screen, friend's hand on the screen) × 2 (target side: left, right) ANOVAs were then carried out for participants' target detection performance in each sitting position. The results showed that when a friend was sitting to the participants' left, there were no significant main effects of hand position, *F*_(2, 54)_ = 1.295, *p* = 0.282, or target side, *F*_(1, 27)_ = 1.107, *p* = 0.302. However, the interaction between hand position and target side was significant, *F*_(2, 54)_ = 3.369, *p* = 0.042. Subsequent paired sample *t*-tests showed that, as in Experiment 1, participants were faster when responding to targets appearing next to their own hand compared to targets appearing away from their hand, *t*_(27)_ = −2.920, *p* = 0.007. However, again there were no differences in detecting targets that were presented near and away from a friend's hand, *t*_(27)_ = 0.508, *p* = 0.615, or when no hand was placed on the screen, *t*_(27)_ = −0.370, *p* = 0.714. Similar results were obtained when a friend was sitting to the participants' right. There were no significant main effects of hand position, *F*_(2, 54)_ = 0.318, *p* = 0.729, or target side, *F*_(1, 27)_ = 0.790, *p* = 0.382. However, the interaction between hand position and target side was significant, *F*_(2, 54)_ = 4.508, *p* = 0.015. Paired-samples *t*-tests showed that participants responded faster to targets appearing next to their hand compared to targets appearing away from their hand, *t*_(27)_ = −2.924, *p* = 0.007. However, this near-hand facilitation effect was absent when a friend's hand was held on the screen, *t*_(27)_ = −0.192, *p* = 0.849, or when no hand was held on the screen, *t*_(27)_ = 1.857, *p* = 0.074. Together, these results suggest that even under conditions in which visual similarity between hands was high, target detection performance was affected by the proximity of one's own but not another's hand.

The results of Experiment 2 again showed that participants responded more quickly to targets appearing near their own hands compared to targets appearing away from their hands, demonstrating the robustness of the attentional bias effect near one's own hand. However, there was no such facilitation effect when comparing the conditions in which participants had to detect targets appearing near or away from their friend's hand. Although we increased the visual similarity between participants' hands and the hands of their friends, the presence of a friend's hand on the screen had no influence on participants' reaction times to detect targets. While visual similarity between an observer's own hand and a fake hand may lead observers to prioritize the space near a fake hand (Reed et al., 2006), the same visual similarity was not sufficient to change visual processing near the hands of another person. Instead, the results of Experiment 2 again point to the conclusion that observers do not prioritize the space near a friend's hand, even when this hand looks like their own.

## Experiment 3

Although Reed et al. (2006) showed that attention is biased to the space near a fake hand when observers wear a rubber glove that matches this false hand, the results of Experiment 2 show that using a similar technique to increase visual similarity between the hands of two people is not sufficient to make observers prioritize the space near the hand of another person. Why would participants show an attentional bias near a fake hand made to look like their own, but disregard the hands of another person that also shared their appearance? One possibility that may explain this discrepancy is that the visual system might not represent a fake hand and a real person's hand in the same way. That is, when observers know they are viewing a hand that belongs to another person, they may treat this hand differently than a hand that looks the same but cannot possibly belong to anyone else. Participants in Reed et al.'s (2006) study who prioritized the space near a fake hand may have incorporated the fake hand into their body schema through the simultaneous tactile sensation of the rubber glove on their own hand combined with the visual signal of the rubber glove on the fake hand. Visual information from a rubber hand that corresponds to an observer's unseen real hand can be sufficient to shift the receptive fields of multisensory neurons (Graziano, 1999; Graziano et al., 2000) and create crossmodal congruency effects (Pavani et al., 2000). In Experiment 3, we examine the idea that a fake hand that is visually similar to an observer's real hand is sufficient to bias attention. We designed this experiment as a replication of Reed et al. (2006) Experiment 4. If participants incorporate a realistic-looking fake hand into their own body schema, then they should show faster detection of targets appearing next to this fake hand.

### Method

#### Participants

Forty-three right-handed North Dakota State University undergraduates (17 females; mean age = 19.50 years) participated in the study for course credit. All had normal or corrected-to-normal vision, and none of them had participated in the previous experiments.

#### Apparatus, procedure, and design

**Figure 5:**
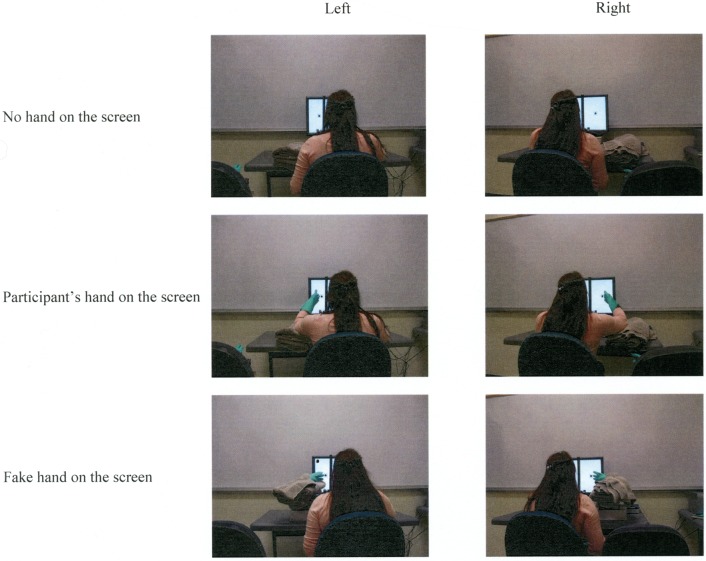
**Experimental setup and hand positions in Experiment 3**.

Experiment 3 was identical to Experiment 2, with the exception that participants were not required to bring a friend to the study; instead, two fake hands were used to replace a friend's left and right hand. As in Experiment 2, when a fake hand was placed on the participants' left, participants had to put a rubber glove on their left hand, and vice versa. The fake hands were made by stuffing rubber gloves with a water bottle and cotton. When a fake hand was placed next to a square on the screen, it was supported by boxes and folded towels on the table. Figure 5 shows the experimental settings and hand positions in the different experimental conditions for Experiment 3.

### Results and discussion

**Figure 6:**
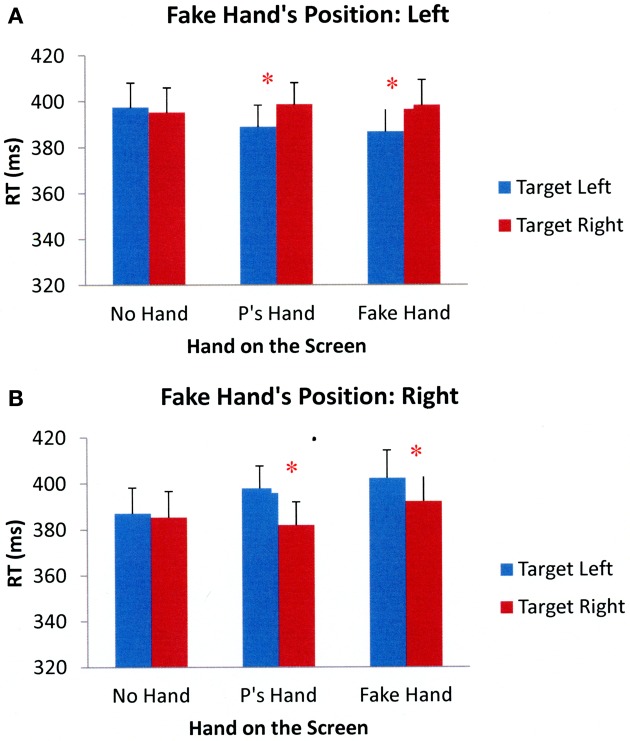
**Mean reaction times across all participants in different Experimental conditions of Experiment 3 when a fake hand was positioned on the participant's left (A) and right (B).** Error bars represent standard errors of the means. (Note: P's = Participant's). ^*^p < 0.05.

Participants' target detection performance was analyzed according to the same criteria as in the previous experiments. Five of the participants were eliminated due to excessive errors on the catch trials. The overall error rate for the remaining participants was 13%^3^. Additionally, data from trials in which participants responded outside the 200–1000 ms window (6% of total trials) were eliminated from analyses. As before, the results of a 2 (fake hand's position: left, right) × 3 (hand position: no hand on the screen, participant's hand on the screen, fake hand on the screen) × 2 (target side: left, right) × 2 (cue validity: valid, invalid) repeated measures ANOVA showed a significant main effect of cue validity, *F*_(1, 37)_ = 171.178, *p* < 0.001, suggesting that participants responded faster to validly cued targets than to invalidly cued targets. There was also a significant interaction between the fake hand's position and target side, *F*_(1, 37)_ = 21.513, *p* < 0.001, and this interaction was affected by hand position, *F*_(2, 74)_ = 7.528, *p* = 0.001. There were no other significant main effects or interactions. The mean RTs across participants in different experimental conditions collapsed across cue validity are shown in Figure 6.

To further examine the significant three-way interaction between the fake hand's position, hand position, and target side, separate 3 (hand position: no hand on the screen, participant's hand on the screen, fake hand on the screen) × 2 (target side: left, right) ANOVAs were performed on participants' target detection performance when a fake hand was positioned on either the left- or right-hand side of the participants. The results showed that when a fake hand was on the participants' left, the main effect of hand position was not significant, *F*_(2, 74)_ = 0.347, *p* = 0.708, but there was a significant main effect of target side, *F*_(1, 37)_ = 5.690, *p* = 0.022. The interaction between hand position and target side was also significant, *F*_(2, 74)_ = 3.631, *p* = 0.031. Subsequent paired sample *t*-tests showed that participants were faster when responding to targets appearing next to their hand compared to targets appearing away from their hand, *t*_(37)_ = −2.404, *p* = 0.021. Participants were also faster when responding to targets that appeared next to the fake hand than to targets that appeared away from the fake hand, *t*_(37)_ = −2.642, *p* = 0.012. However, there were no performance differences in target detection when no hand was placed on the screen, *t*_(37)_ = 0.519, *p* = 0.607. Similar results were obtained when a fake hand was on participants' right. The main effect of hand position was not significant, *F*_(2, 74)_ = 2.074, *p* = 0.133, but there was a significant main effect of target side, *F*_(1, 37)_ = 14.651, *p* < 0.001 and the interaction between hand position and target side was also significant, *F*_(2, 74)_ = 3.269, *p* = 0.044. Paired-sample *t*-tests showed that participants had faster RTs to targets that appeared near their hand than to targets that appeared away from their hand, *t*_(37)_ = −3.912, *p* < 0.001. Participants also responded faster to targets that appeared next to the fake hand compared to targets that appeared away from the fake hand, *t*_(37)_ = −2.928, *p* = 0.006. There were no differences in target detection times when no hand was held on the screen, *t*_(37)_ = 0.409, *p* = 0.685. Together, the results suggest that visual attention can be biased by the proximity of one's own hand as well as a visually similar fake hand.

Participants in Experiment 3 were faster to respond to targets appearing near a fake hand compared to targets appearing away from a fake hand, replicating previous research (Reed et al., 2006) showing that observers prioritize the space near not only their own hands, but also fake hands. These results suggest that people may prioritize the space near a fake hand because they represent the fake hand in multisensory areas. Visual information about hand position provided by a fake hand is sufficient to facilitate responses to objects appearing near the hand. However, although the visual information available to participants in the friend's hand conditions of Experiment 2 was quite similar to the visual information available in the fake hand conditions of the current experiment, the results of Experiment 2 suggest that the visual system treats a real person's hand differently than a fake rubber hand, showing no attentional bias to the real person's hand. Participants may have more readily incorporated a fake hand into their own body schema than their friends' hands: although some evidence suggests that observers incorporate fake hands into their own body schema in the absence of synchronous feedback between visual signals and tactile sensations (e.g., Pavani et al., 2000; Durgin et al., 2007), experiences of confusing another person's hand for one's own typically involve direct synchrony between what observers see and feel (Tsakiris et al., 2005; Schütz-Bosbach et al., 2006), more synchrony than was provided by the conditions of Experiment 2.

The results of the first three experiments suggest that observers may only experience altered vision near the hands when they have incorporated these hands into their own body schema. In Experiments 1 and 2, when participants' friends sat passively throughout the entire experiment, never performing any actions that were relevant to the participants' task, participants showed no visual biases associated with the presence of their friends' hands on the display. The friends were distinctly separate from the participants and the task they were asked to perform.

However, previous studies have shown that external objects can be integrated into one's own body schema after a short period of tool-use training (e.g., Iriki et al., 1996; Maravita et al., 2002; Maravita and Iriki, 2004; Cardinali et al., 2009) and that such tool use can also drive changes in visual processing (e.g., Tseng et al., 2012; Brockmole et al., 2013). Recent findings also suggest that when two people work together on a task, they likewise incorporate representations of their partner's task-relevant body parts into a joint body-schema (Soliman et al., 2012, in preparation). If this is the case, then we predict that attention should be facilitated by the presence of another's hand after participants perform a cooperative task which can enhance the incorporation of another's body parts into their own body schemas. We tested this prediction in Experiment 4.

## Experiment 4

The results of Experiments 1–3 showed that participants do not by default prioritize the space near their friend's hand for attention. Here we investigate the hypothesis that observers will show a bias for the space near another person's hands when they first work together on a joint action task. This task is designed to induce participants to develop a joint body-schema (Soliman et al., 2012, in preparation), essentially serving the same purpose as tool-use training periods in experiments showing that actors integrate tools into their body schemas (e.g., Iriki et al., 1996). In the task, we asked participants and their friends to each hold the end of a wire in one hand and to work together to saw through a wax block using this wire. Following this “training” period, participants again performed the covert-orienting task. If a shared body representation plays an influential role in biasing attention to another's hand, then participants should show better detection performance when the target appears near their own hand as well as near another person's hand after the joint wax-sawing task. However, if a shared body representation is not sufficient for participants to prioritize the space near another's hand for attention, then they should show faster target detection when the targets are presented near their own but not another's hand after the joint wax-sawing task.

### Method

#### Participants

Thirty-five right-handed North Dakota State University undergraduates (28 females; mean age = 19.43 years) participated in the study for course credit with a same-sex friend. All had normal or corrected-to-normal vision and had not participated in the previous experiments.

#### Apparatus, procedure, and design

**Figure 7:**
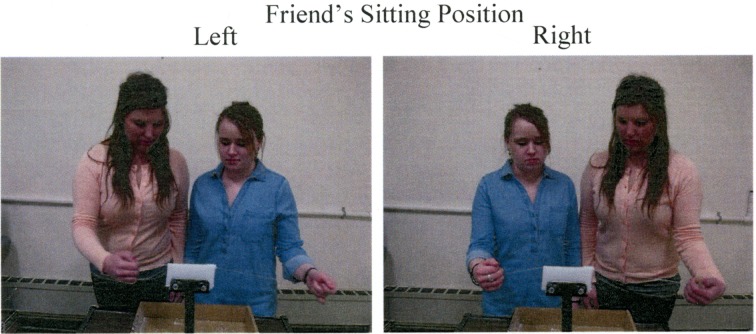
**Experimental setup for the joint wax-sawing task prior to the Posner cueing task in Experiment 4**.

Experiment 4 was identical to Experiment 1, with the exception that participants and their friends had to perform a joint wax-sawing task for 4 min before the Posner cueing task for each friend's sitting position. Participants and their friends were asked to use a wire to saw a wax block that was held in a holder attached to a table (cf. Soliman et al., 2012, in preparation). The participant held the end of a wire in one hand while the friend held the other end of the wire in the opposite hand. To complete this task, both parties had to coordinate their actions; as participants pulled the wire toward themselves, their friends had to push the wire away from themselves and vice-versa. For the condition in which a friend's sitting position was on the left, the wax-sawing task was performed while the friend stood on the left side of the wax holder using the left hand to saw and the participant stood on the right using the right hand to saw. For the condition in which a friend's sitting position was on the right, participants and their friends switched positions and hands for the wax-sawing task such that the hand the friend used to saw was also the hand they would place on the display. Wax blocks were replaced as needed to keep participants sawing for 4 min. Figure 7 shows the experimental setup for the joint wax-sawing task.

### Results and discussion

**Figure 8:**
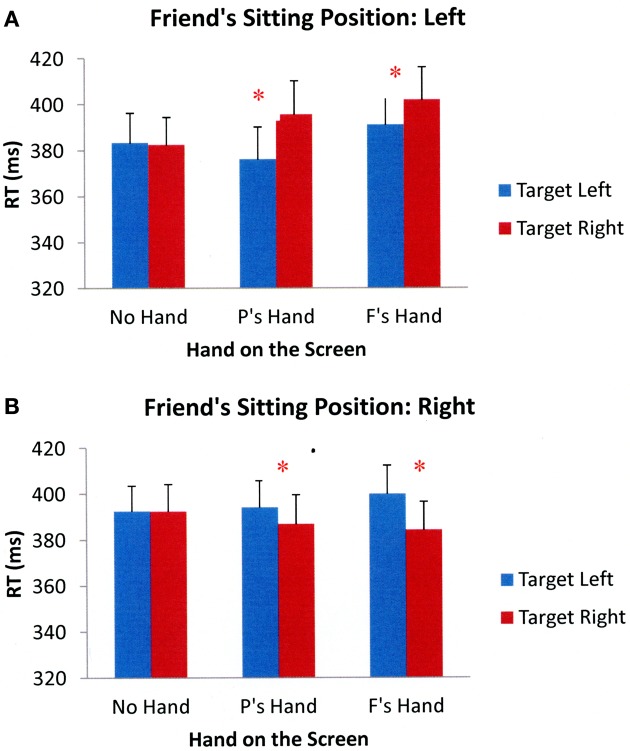
**Mean reaction times across all participants in different Experimental conditions of Experiment 4 when a friend was sitting to the participant's left (A) and right (B).** Error bars represent standard errors of the means. (Note: P's = Participant's; F's = Friend's). ^*^p < 0.05.

Participants' target detection performance was analyzed according to the same criteria as in the previous experiments. Two participants were excluded due to excessive errors on the catch trials. For the remaining participants, the overall error rate on catch trials was 11%^4^. Additionally, 5% of the total trials were eliminated for falling outside the 200–1000 ms response window. As before, the results of a 2 (friend's sitting position: left, right) × 3 (hand position: no hand on the screen, participant's hand on the screen, friend's hand on the screen) × 2 (target side: left, right) × 2 (cue validity: valid, invalid) repeated measures ANOVA showed a significant main effect of cue validity, *F*_(1, 32)_ = 122.364, *p* < 0.001, showing that participants responded faster to validly cued targets than to invalidly cued targets. There was also a significant interaction between friend's sitting position and hand position, *F*_(2, 64)_ = 3.563, *p* = 0.034, as well as a significant interaction between friend's sitting position and target side, *F*_(1, 32)_ = 22.494, *p* < 0.001. The interaction between hand position and target side was also significant, *F*_(2, 64)_ = 3.324, *p* = 0.042. More importantly, the interaction between friend's sitting position, hand position, and target side was also significant, *F*_(2, 64)_ = 9.434, *p* < 0.001. There were no other significant main effects or interactions. Figure 8 shows the mean RTs across participants in different experimental conditions collapsed across cue validity.

To further examine the significant three-way interaction between friend's sitting position, hand position, and target side, separate 3 (hand position: no hand on the screen, participant's hand on the screen, friend's hand on the screen) × 2 (target side: left, right) ANOVAs were performed for each friend's sitting position. When a friend was sitting to the left-hand side of the participant, the results showed significant main effects of hand position, *F*_(2, 64)_ = 3.783, *p* = 0.028, and target side, *F*_(1, 32)_ = 11.477, *p* = 0.002. However, the interaction between hand position and target side was also significant, *F*_(2, 64)_ = 7.652, *p* = 0.001. Subsequent paired sample *t*-tests showed that participants were faster when responding to targets appearing next to their hand compared to targets appearing away from their hand, *t*_(32)_ = −4.096, *p* < 0.001. Additionally, participants were faster when responding to targets that appeared next to their friend's hand than to targets that appeared away from their friend's hand, *t*_(32)_ = −2.696, *p* = 0.011. There were no performance differences in target detection when no hand was placed on the screen, *t*_(32)_ = 0.277, *p* = 0.783. When a friend was sitting on the right-hand side of the participants, the results showed no effect of hand position, *F*_(2, 64)_ = 0.108, *p* = 0.897, but the main effect of target side was significant, *F*_(1, 32)_ = 6.310, *p* = 0.017. The interaction between hand position and target side was also significant, *F*_(2, 64)_ = 5.192, *p* = 0.008. Paired-samples *t*-tests showed that participants had faster RTs to targets that appeared near their hand than to targets that appeared away from their hand, *t*_(32)_ = −2.040, *p* = 0.050. Participants also responded faster to targets that appeared next to a friend's hand compared to targets that appeared away from a friend's hand, *t*_(32)_ = −4.114, *p* < 0.001. There were no differences in target detection times when no hand was held on the screen, *t*_(32)_ = 0.018, *p* = 0.985. Together, the results suggest that visual attention can be biased both by the proximity of participants' own hands and their friends' hands after performing a joint action task. When participants have a reason to incorporate a representation of a friend's hands into their own body schema, their visual systems show altered processing near these hands.

## General discussion

The main purpose of the present study was to investigate how observers allocate attention to visual information presented not only near their own hands, but also near the hands of other actors. Across all four experiments, participants consistently detected targets appearing near their own hands more quickly than targets appearing away from their hands. Additionally, participants in Experiment 3 detected targets more quickly when the targets were presented near a fake hand than when the targets were presented away from a fake hand. The results of Experiments 1 and 2 demonstrate that the mere presence of another's hand was not sufficient to bias attention to the space near this hand: participants in these experiments were no faster to detect targets that appeared close to their friend's hand than targets appearing away from the friend's hand. However, in Experiment 4, after the participants and their friend performed a joint action task together, participants were faster to detect targets appearing near the friend's hand than targets appearing away from the friend's hand.

Across experiments, the present study robustly shows that target detection is faster when targets are presented near participants' hands than when targets are presented away from their hands. These results are consistent with previous work from Reed et al. (2006, 2010), backing up the notion that attention is prioritized for the space near the hand. Objects appearing within perihand space present opportunities for interaction and affect visual processing. This altered vision near the hands may arise via populations of bimodal visuotactile neurons responding exclusively to visual and tactile stimuli presented near or on the hand (Graziano and Gross, 1993, 1998) that strengthen object processing in the space near the hand (e.g., Graziano et al., 1997; di Pellegrino and Frassinetti, 2000; Schendel and Robertson, 2004; Reed et al., 2006, 2010; Abrams et al., 2008; Cosman and Vecera, 2010).

We hypothesized that since observers are sensitive to the signals generated by others' hands (e.g., Langton et al., 1996; Sebanz et al., 2006; Fischer et al., 2008) and experience motor resonance when watching others act (e.g., Rizzolatti and Craighero, 2004), they might also show biases in processing objects presented near the hands of other actors. However, this was not the case in the present study. Our results indicate that observers do not by default prioritize the space near another person's hand. In Experiments 1 and 2, although participants showed a bias toward targets appearing near their own hands, they showed no differences in detecting targets appearing near or away from another's hand. Note that this finding cannot be explained by the lack of proprioceptive information about one's own hand being on the screen: in Experiment 3, even when participants' own hands were in their laps, their attention was biased toward targets appearing near a fake hand. We propose that this facilitation of detection near a fake hand was a result of participants incorporating the fake hand into their own body schema. In other words, participants detected targets that were near the fake hand more quickly because they represented these items as appearing in perihand space.

The results of Experiment 4 further strengthen our inference on the necessity of an observer incorporating a hand into her own body schema in order to experience altered vision near this hand. In Experiment 4, participants' target detection performance was significantly improved for targets appearing near their friend's hand after a joint action task, presumably because the joint action task facilitated the incorporation of another person's hand into one's own body schema. Participants only showed biased attention to the space near their friends' hands after these hands became relevant to accomplishing a shared goal. The results are consistent with a recent study conducted by Soliman et al. (2012, in preparation) showing that after performing a rhythmic sawing task with a partner, participants were slower to localize a vibration applied to their fingers when spatially incongruent visual stimuli (LEDs) were simultaneously observed near the partner's fingers. The researchers attribute the enhanced interference effect to participants incorporating the partner's collaborating hand into their own body representations (Soliman et al., 2012, in preparation). Similarly, we find that participants respond to a visual stimulus appearing near a friend's hand as if it were their own only after engaging in a cooperative task with the friend.

In addition to shedding new light on the question of how the presence of another's hands influences visual processing, our results also add to the growing literature demonstrating the plasticity of body representations. The fact that participants showed biased processing near a friend's hand in Experiment 4 are in line with previous research showing that observers incorporate used tools into the body schema to extend representation of peripersonal space (e.g., Iriki et al., 1996; Maravita et al., 2002; Maravita and Iriki, 2004; Cardinali et al., 2009, 2012). Our findings also compliment those of Reed et al. (2010) in which participants were faster to detect targets appearing near the prongs of a small rake following practice using this tool and research showing that observed tool use can affect perception (Bloesch et al., 2012). Taken together, our results, along with the many others mentioned above, suggest that the representation of one's own body is flexible and that external objects such as tools or even the hands of another actor may be incorporated into one's own body schema following relevant practice or training. These findings also lend support to the notion that changes in visual processing near the hands and the ends of tools are driven by bimodal neurons (Reed et al., 2006, 2010): observers only seem to prioritize the space near a hand or tool when it has been incorporated into the body schema and presumably receives representation in multisensory areas of the brain.

In conclusion, the present study further solidifies the claim that observers prioritize the space near their own hands. In addition, we find that observers do not by default experience changes in visual processing near the hands of other people. However, following a cooperative joint action task, participants show a bias for detecting targets not only near their own hands, but also near the hands of the other actor. These findings suggest that shared body representations may play a crucial role in generating visual biases near the hands of other actors.

### Conflict of interest statement

The authors declare that the research was conducted in the absence of any commercial or financial relationships that could be construed as a potential conflict of interest.

## Footnotes

^1^We also conducted a 2 (friend's sitting position: left, right) × 3 (hand position: no hand on the screen, participant's hand on the screen, friend's hand on the screen) repeated measures ANOVA on catch trial error data that revealed a significant main effect of the friend's sitting position, *F*_(1, 29)_ = 8.108, *p* = 0.008, showing that participants made more catch-trial errors when a friend sat on their left-hand side (20%) compared to when a friend sat on their right-hand side (13%). The speed advantage for responses when a friend sat on the left-hand side may therefore also reflect a speed-accuracy trade off. No other main effects or interactions were significant for the error data (all *p*-values >0.05).

^2^An analysis of catch trial errors revealed no significant main effects or interactions between the factors of friend's sitting position and hand position (all *p*-values >0.05).

^3^An analysis of catch trial errors showed no significant main effects or interactions (all *p*-values >0.05) between friend's sitting position and hand position, indicating no systematic effects of these factors on errors.

^4^As in the previous experiments, an ANOVA on catch trial errors with factors of friend's sitting position and hand position showed no significant main effects or interactions (all *p*-values >0.05), indicating no systematic effects of these factors on catch trial errors.
